# Blockade of Rostral Ventrolateral Medulla (RVLM) Bombesin Receptor Type 1 Decreases Blood Pressure and Sympathetic Activity in Anesthetized Spontaneously Hypertensive Rats

**DOI:** 10.3389/fphys.2016.00205

**Published:** 2016-06-02

**Authors:** Izabella S. Pinto, Aline A. Mourão, Elaine F. da Silva, Amanda S. Camargo, Stefanne M. Marques, Karina P. Gomes, James O. Fajemiroye, Angela A. da Silva Reis, Ana C. S. Rebelo, Marcos L. Ferreira-Neto, Daniel A. Rosa, André H. Freiria-Oliveira, Carlos H. Castro, Eduardo Colombari, Diego B. Colugnati, Gustavo R. Pedrino

**Affiliations:** ^1^Department of Physiological Sciences, Center for Neuroscience and Cardiovascular Research, Biological Sciences Institute, Federal University of GoiásGoiânia, Brazil; ^2^Postgraduate Programme in Pharmaceutical Sciences, Faculty of Pharmacy, Federal University of GoiásGoiânia, Brazil; ^3^Department of Biochemistry and Molecular Biology, Biological Sciences Institute, Federal University of GoiásGoiânia, Brazil; ^4^Department of Morphology, Biological Sciences Institute, Federal University of GoiásGoiânia, Brazil; ^5^Department of Physiology, College of Physical Education, Federal University of UberlândiaUberlândia, Brazil; ^6^Department of Physiology and Pathology, School of Dentistry, São Paulo State UniversityAraraquara, Brazil

**Keywords:** rostral ventrolateral medulla, bombesin, BB_1_ receptors, BIM-23127, SHR

## Abstract

Intrathecal injection of bombesin (BBS) promoted hypertensive and sympathoexcitatory effects in normotensive (NT) rats. However, the involvement of rostral ventrolateral medulla (RVLM) in these responses is still unclear. In the present study, we investigated: (1) the effects of BBS injected bilaterally into RVLM on cardiorespiratory and sympathetic activity in NT and spontaneously hypertensive rats (SHR); (2) the contribution of RVLM BBS type 1 receptors (BB_1_) to the maintenance of hypertension in SHR. Urethane-anesthetized rats (1.2 g · kg^−1^, i.v.) were instrumented to record mean arterial pressure (MAP), diaphragm (DIA) motor, and renal sympathetic nerve activity (RSNA). In NT rats and SHR, BBS (0.3 mM) nanoinjected into RVLM increased MAP (33.9 ± 6.6 and 37.1 ± 4.5 mmHg, respectively; *p* < 0.05) and RSNA (97.8 ± 12.9 and 84.5 ± 18.1%, respectively; *p* < 0.05). In SHR, BBS also increased DIA burst amplitude (115.3 ± 22.7%; *p* < 0.05). BB_1_ receptors antagonist (BIM-23127; 3 mM) reduced MAP (–19.9 ± 4.4 mmHg; *p* < 0.05) and RSNA (−17.7 ± 3.8%; *p* < 0.05) in SHR, but not in NT rats (−2.5 ± 2.8 mmHg; −2.7 ± 5.6%, respectively). These results show that BBS can evoke sympathoexcitatory and pressor responses by activating RVLM BB_1_ receptors. This pathway might be involved in the maintenance of high levels of arterial blood pressure in SHR.

## Introduction

Bombesin (BBS), a tetradecapeptide isolated from the skin of the frog *Bombina bombina* (Anastasi et al., 1971), have shown broad spectrum of biological activities (Brown, 1983; Gonzalez et al., 2008; Jensen et al., 2008). The BBS activates three G protein-coupled receptors: bombesin receptor 1 (BB_1_), bombesin receptor 2 (BB_2_), and bombesin receptor 3 (BB_3_). BBS-like peptides—Neuromedin B (NB) and gastrin releasing peptide (GRP) are natural ligand of the BB_1_ and BB_2_ receptors, respectively (Jensen et al., 2008). Natural agonist of the BB_3_ receptor still remains unknown. However, it seems that BB_3_ receptor plays an important physiological role, since BB_3_ receptor *knockout* mice developed obesity associated with hypertension and impairment of glucose metabolism (Ohki-Hamazaki et al., 1997). In human, the BB_1_ receptor gene is at chromosome 6p21-pter (Jensen et al., 2008). The BB_1_ receptor signal occurs primarily through phospholipase-C-mediated cascades, that involve activation of G_q_α protein and consequent stimulation of protein kinase C (Jensen et al., 2008).

In mammals, BBS receptors and BBS-like peptides are distributed in the Central Nervous System (CNS) (Woodruff et al., 1996; Jensen et al., 2008) including regions involved in the cardiorespiratory control (Chung et al., 1989; Lynn et al., 1996; Li et al., 2016). The administration of BBS has been reported to enhancebreathing (Holtman et al., 1983; Glazkova and Inyushkin, 2006), raise plasma concentration of catecholamine (Brown and Fisher, 1984), tachycardia (Zogovic and Pilowsky, 2011), increase blood pressure (Brown, 1983; Zogovic and Pilowsky, 2011), and sympathetic tone (Zogovic and Pilowsky, 2011) in normotensive (NT) rats. Zogovic and Pilowsky (2011) showed that intrathecal injection of BBS is associated with sympathoexcitatory and pressor responses. In the same study, the authors also reported that the administration of an antagonist of BBS receptor 2 attenuated the effects of BBS on blood pressure of NT rats. However, the involvement of the rostral ventral medulla (RVLM) in the BBS-induced cardiorespiratory and autonomic responses as well as the contribution of the BBS receptor type 1 to the maintenance of blood pressure in NT rats and spontaneously hypertensive rats (SHR) is still unclear.

The RVLM contains neurons that regulate peripheral sympathetic vasomotor tone and blood pressure (Guertzenstein, 1973; Guertzenstein and Silver, 1974; Guyenet et al., 1989; Guyenet, 2006; Toney and Stocker, 2010). The RVLM is localized ventral to the rostral part of the nucleus ambigus (NA), caudal to the facial nucleus and ventral to the Bötzinger complex. The RVLM neurons project to the sympathetic preganglionic neurons located in the intermediolateral (IML) cell column of the spinal cord (Loewy, 1981; Millhorn and Eldridge, 1986; Guyenet, 2006). The neurons projecting from RVLM could modulate peripheral sympathetic activity to the kidneys, vessels, heart, and adrenal gland.

The hyperactivity of RVLM neurons has been implicated in the maintenance of hypertension in different experimental models (Yang et al., 1995; Fink, 1997; Ito et al., 2000, 2003; Matsuura et al., 2002; Adams et al., 2007; Stocker et al., 2007; Toney and Stocker, 2010). Previous studies have shown that the injection of excitatory amino acid (EAA) antagonist into the RVLM reduced arterial pressure in SHR but not in NT rats (Ito et al., 2000). In addition, the electrophysiological studies have shown that firing rate of RVLM neurons is significantly faster in neonatal and adult SHR than NT rats (Chan et al., 1991; Matsuura et al., 2002). These findings indicate that hyperactivity of the RVLM neurons could contribute to the development and maintenance of hypertension in SHR.

Hence, we hypothesized whether or not the injection of BBS into RVLM affects cardiorespiratory and sympathetic activities in NT rats and SHR. In order to test this hypothesis, the recording of the mean arterial pressure (MAP), renal sympathetic nerve activity (RSNA) and diaphragm (DIA) motor activity were carried out to evaluate changes induced by unilateral and bilateral injection of BBS into RVLM of urethane-anesthetized NT rats and SHR. In addition, the contribution of tonic activation of BBS receptor 1 in the maintenance of high levels of arterial pressure in SHR was also evaluated.

## Methods

### Animals and ethical approval

Male Wistar NT rats and SHR weighing 250 to 330 g were used. Animals were housed in a temperature-controlled room (22–24°C) with a 12:12-h light-dark cycle (lights on at 07:00), free access to food, and tap water. All experimental procedures were designed in strict adherence to the National Health Institute Guidelines for Care and Use of Laboratory Animals, and approved by Ethics Committee for Animal Care and Use (CEUA) of the Federal University of Goiás (number of ethical committee: 025/12).

### Surgical procedures

Rats were anesthetized with halothane (2–3% in O_2;_ Tanohalo; Cristália, SP, Brazil). The right femoral vein and artery were catheterized for drug administration and blood pressure recording, respectively. After catheterization, anesthesia was maintained by intravenous administration of urethane (1.2 g · kg^−1^ b.wt.; Sigma–Aldrich, St. Louis, MO, USA). The trachea was cannulated to reduce airway resistance. Bipolar stainless steel electrodes were implanted in the DIA muscle for electromyography (EMG) recording of inspiratory motor activity. Rats were later mounted prone in a stereotaxic apparatus for craniotomy and instrumented for the recording of RSNA. The body temperature of rats was maintained at 37 ± 0.5°C with a thermostatically controlled heated table.

### Recording of cardiorespiratory parameters

In order to record the arterial pressure, the arterial catheter was connected to a pressure transducer which is coupled to an amplifier (Bridge Amp FE221; ADInstruments, Colorado Springs, CO, USA). The pulsatile pressure was recorded continuously with a data acquisition system (PowerLab; ADInstruments, Colorado Springs, CO, USA). The MAP was calculated from the pulsatile signal using the LabChart software (v.7.3.7, ADInstruments, Colorado Springs, CO, USA). Analogical signals of the electrocardiogram (ECG), obtained through electrodes positioned in the forelimbs, were amplified 1000 times and filtered between 100 and 1000 Hz (Bridge Amp; ADInstruments, Colorado Springs, CO, USA). The heart rate (HR) was calculated as instantaneous frequency of the ECG signal (LabChart v.7.3.7, ADInstruments, Colorado Springs, CO, USA). The DIA motor activity signals was amplified 10,000 times (Bridge Amp; ADInstruments, Colorado Springs, CO, USA) and band-pass filtered (100–2000 Hz). The signal were rectified and integrated in 50 ms intervals using LabChart software (v.7.3.7; ADInstruments, Colorado Springs, CO, USA). The DIA motor activity was evaluated by burst amplitude (expressed as percentage difference from baseline) and frequency (considered as respiratory frequency, fR, and expressed in cycles per minute, cpm).

### Recording of renal sympathetic nerve activity

RSNA was recorded through the left renal nerve with bipolar silver electrodes. The renal nerve was located, dissected and covered with mineral oil prior to the placement of electrodes for recording (Nujol - Schering-Plough, São Paulo, SP, Brazil). The signals were obtained using a high-impedance probe connected to the amplifier (P511; Grass Instruments, Quincy, MA, USA). The signal was amplified 20,000 times, digitized and band-pass filtered (30–1000 Hz). The nerve signal was recorded continuously (with a PowerLab System-ADInstruments; Colorado Springs, CO, USA), rectified and integrated at 1 s intervals using LabChart software (v.7.3.7.; ADInstruments; Colorado Springs, CO, USA). At the end of each experiment, ganglionic blocker hexamethonium (30 mg · kg^−1^, b.wt., i.v.; Sigma–Aldrich, St. Louis, MO, USA) was administered to determine the background noise. The level of RSNA was expressed as a percentage of baseline after subtraction of the noise.

### Respiratory synchronization

Analyses of the respiratory synchronization were made offline using Spike2 software (version 8; Cambridge Electronic Design Limited, Cambridge, CAM, England). In order to analyze respiratory modulation, RSNA was rectified and signals were smoothed using a time constant of 50 ms. The DIA-triggered averages of RSNA were generated after nanoinjection of vehicle and bilateral BBS into RVLM. Averages of RSNA were made using 15 DIA burst as trigger events. The time of inspiration was determined based on the duration of the inspiratory DIA burst while expiratory time was determined between consecutive DIA burst. The RSNA post-inspiratory peak was later evaluated.

### Nanoinjections into RVLM

Animals were mounted prone in a stereotaxic apparatus (David Kopf Instruments, Tujunga, CA, USA) with incisor bar 11 mm below the interaural line. After partial removal of the occipital bone, the meninges covering the dorsal surface of the brainstem was opened up surgically to visualize the *calamus scriptorius*. In order to nanoinject into the RVLM, a glass micropipette was positioned as follows: 2.5 mm rostral from the *calamus scriptorius*, ±2.0 mm lateral from the midline and 2.5 mm ventral from the dorsal surface.

Firstly, RVLM was localized through unilateral nanoinjection (50 nl) of L-glutamate (10 mM; Sigma-Aldrich, St. Louis, MO, USA) in all experimental groups. RVLM was considered previously identified when L-glutamate nanoinjection increased MAP in approximately 20 mmHg. After the return of cardiorespiratory and sympathetic activities to basaline, the bilateral nanoinjection (50 nl each) of vehicle (150 mM NaCl; Sigma-Aldrich, St. Louis, MO, USA), BBS (0.3 mM; Bachem AG, Bubendorf, Switzerland) or bombesin receptor 1 (BB_1_) antagonist (3 mM; [D-2- NaI^5^-Cys^6, 11^-Tyr^7^,D-Trp^8^,Val^10^,2-NaI^12^-Somatostatin-14 (5–12) amide trifluoroacetate salt]; BIM-23127; Bachem AG, Bubendorf, Switzerland) was injected into RVLM. In order to confirm the integrity of RVML, L-glutamate (10 mM; 50 nl) was nanoinjected again. This nanoinjection produced similar increase in MAP (~20 mmHg) as previously observed in the initial L-glutamate nanoinjection.

### Histology

At the end of the experiment, 2% Evans Blue solution (50 nl; Sigma-Aldrich, St. Louis, MO, USA) was nanoinjected bilaterally into RVLM for histological analyses with the aim of confirming the accuracy of injection sites. Rats were perfused transcardially with saline (150 mM NaCl; 300 mL), followed by 10% formaldehyde (300 mL; Synth Ltda, Diadema, SP, Brazil). The brains were later removed and fixed in 10% formaldehyde. Frozen brains were cut into 40 μm coronal sections and stained with 1% neutral red to determine the nanoinjection sites.

### Experimental protocols

The MAP, DIA motor activity and RSNA were recorded (NT, *n* = 7; SHR, *n* = 7). After a period of stabilization, bilateral nanoinjections of BBS (0.3 mM in 50 nl each) or equivalent volume of vehicle (150 mM NaCl in 50 nl each) was nanoinjected into RVLM. In another group of NT rats (*n* = 6) and SHR (*n* = 6), BIM-23127 (BB_1_ receptor antagonist; 3 mM in 50 nl each) was nanoinjected bilaterally into RVLM. In order to confirm the blockade of BB_1_ receptors, in separated groups of NT rats (*n* = 3) and SHR (*n* = 6), unilateral nanoinjection of BBS (0.3 mM in 50 nl each) 15 min before and 15 min after BIM-23127 (3 mM in 50 nl each) was carried out.

### Statistical analysis

The GraphPad Prism software (v.6; GraphPad Software, Inc., La Jolla, CA, USA) was used for the statistical analysis of experimental data. The basal values and changes in the respiratory synchronization induced by BBS nanoinjection were compare between the groups using an unpaired and paired Student's *t*-test. The autonomic and cardiovascular effects induced by nanoinjection of vehicle, BBS, and BIM into the RVLM were analyzed using two-way ANOVA prior to the Newman–Keuls test. The value of *p* < 0.05 was considered statistically significant.

## Results

### Histological analysis

**Figure 1:**
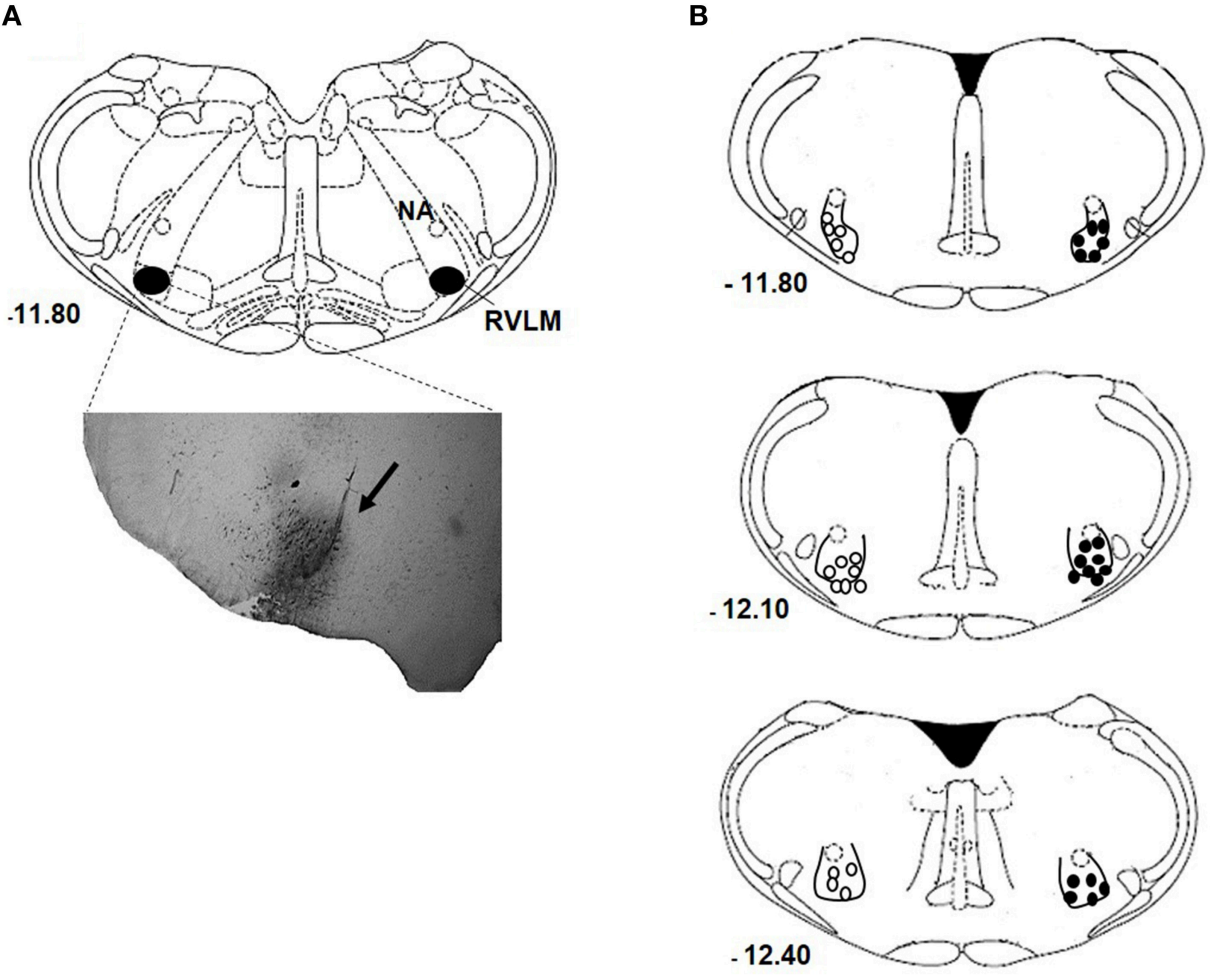
**RVLM nanoinjection site**. Photomicrograph of a coronal section of the medulla showing a representative site of injection labeled with 4% Evans Blue dye into RVLM (arrow; **A**). Schematic drawing showing the center of the nanoinjection site to the bregma in normotensive (open circles) and hypertensive (closed circles) rats nanoinjected with either bombesin (0.3 mM) or BIM-23127 (3 mM; **B**), and rats that received unilateral nanoinjection of bombesin before and after BIM-23127. NA, nucleus ambiguous.

Figure 1A show a representative photomicrograph of the brainstem section that indicates the accuracy of nanoinjection site—RVLM. The center of the nanoinjection site distributions at rostral and caudal levels of RVLM are shown in Figure 1B. Only animal with RVLM confined nanoinjections were analyzed.

### Cardiorespiratory and sympathetic changes produced by the injections of BBS into RVLM

Table 1 shows the body weight and basal values of MAP, RSNA, and fR of NT rats and SHR. There were no changes in RSNA and body weight among groups. However, higher MAP and lower fR were observed in SHR when compared with NT rats (*p* < 0.05).

**Table 1:** **Body weight (b.w) and basal values of mean arterial pressure (MAP), renal sympathetic nerve activity (RSNA), and respiratory frequency (fR) of normotensive (NT) and spontaneously hypertensive rats (SHR) that received nanoinjections of bombesin (BBS; 0.3 mM) or BIM-23127 (3 mM) into RVLM**.

**Groups**	**Injection**	**b.w (g)**	**MAP (mmHg)**	**RSNA (a.u.)**	**fR (cpm)**
NT I	BBS	294.7 ± 4.9	86.3 ± 5.6	0.052 ± 0.02	95.9 ± 3.1
NT II	BIM-23127	287.1 ± 6.7	92.4 ± 5.1	0.061 ± 0.01	96.1 ± 6.5
NT III	BBS-BIM-BBS	284 ± 4.2	108.1 ± 8.9	0.123 ± 0.01	102.8 ± 11.4
SHR I	BBS	275.7 ± 3.8	127.6 ± 2.1^*^	0.107 ± 0.02	66.0 ± 1.9^*^
SHR II	BIM-23127	288.1 ± 7.1	126.6 ± 3.1^*^	0.163 ± 0.05	69.3 ± 3.4^*^
SHR III	BBS-BIM-BBS	291.1 ± 5.2	127.0 ± 2.9^*^	0.110 ± 0.02	70.8 ± 5.5^*^

*Values are expressed as mean ± SEM*.

* *different from normotensive groups; p < 0.05. n = 3–7*.

**Figure 2:**
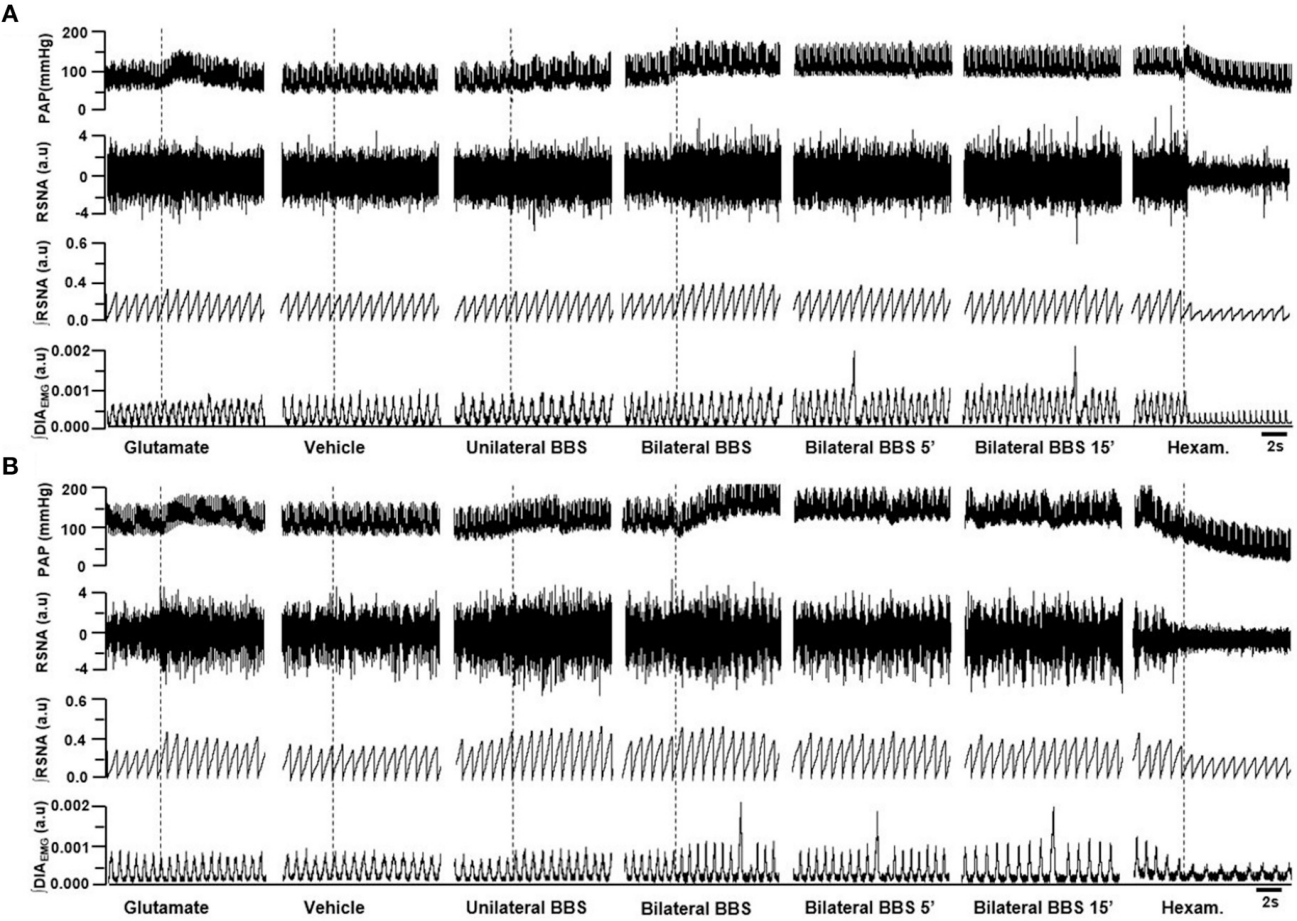
**Examples showing the cardiorespiratory and sympathetic effects induced by BBS into RVLM**. Digitized record of cardiovascular, sympathetic, and respiratory responses produced by unilateral nanoinjections of L-glutamate (10 mM; 50 nl), vehicle (150 mM NaCl), unilateral bombesin (BBS; 0.3 mM), bilateral bombesin (BBS; 0.3 mM; 1, 5, and 15 min) into RVLM of normotensive **(A)** and hypertensive rats **(B)**. Pulsatile arterial pressure (PAP), renal sympathetic nerve activity (RSNA), integrate of renal sympathetic nerve activity (∫RSNA), integrate of diaphragm motor activity (∫DIA_EMG_) and hexam (hexamethonium).

**Figure 3:**
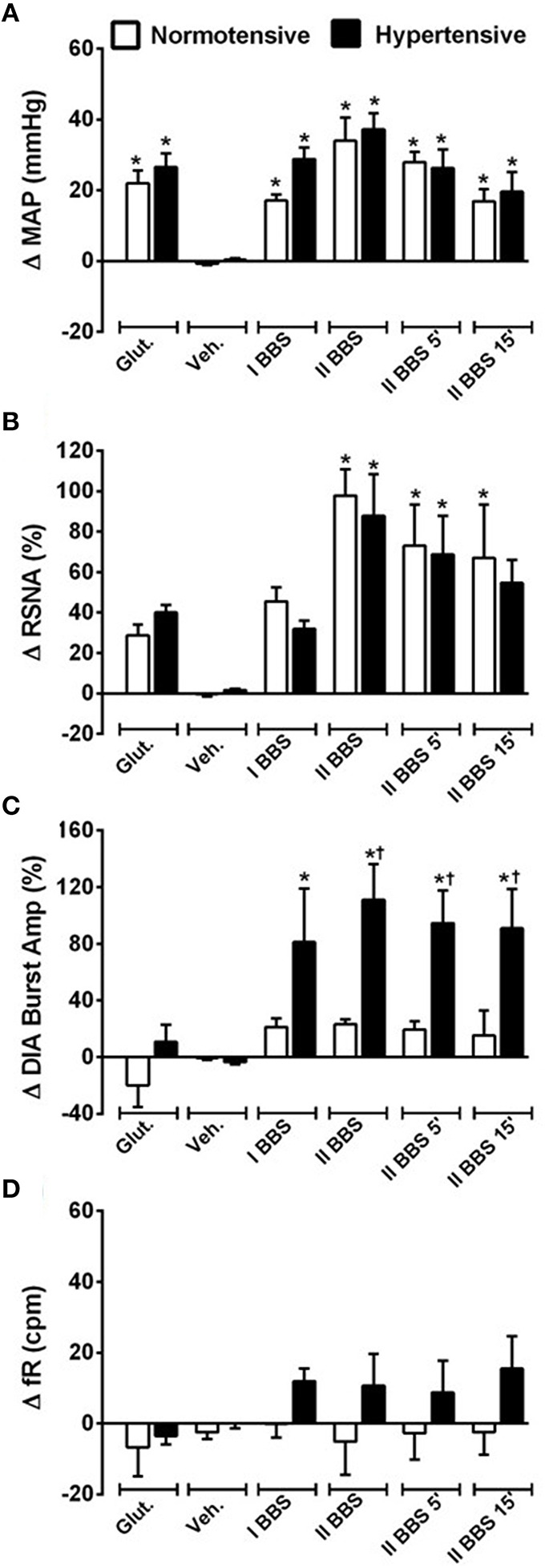
**Cardiovascular, sympathetic, and respiratory responses induced by bombesin naoinjected into RVLM**. Changes induced by unilateral nanoinjecton of L-glutamate (Glut, 10 mM; 50 nl), vehicle (Veh, 150 mM NaCl), bombesin (I BBS, 0.3 mM), and bilateral nanoinjection of bombesin (II BBS; 0.3 mM; 1, 5, and 15 min) into RVLM on mean arterial pressure (MAP; **A**), renal sympathetic nerve activity (RSNA; **B**), diaphragm burst amplitude (DIA Burst Amp; **C**) and respiratory frequency (fR; **D**) in normotensive and hypertensive rats. ^*^different from vehicle; ^†^different from normotensive rats. *p* < 0.05; *n* = 7.

Unilateral nanoinjection of L-glutamate into RVLM increased MAP in both NT rats (ΔMAP: 21.6 ± 5.6 mmHg vs. vehicle −0.7 ± 0.5 mmHg; *p* < 0.05) and SHR (26.3 ± 6.3 mmHg vs. vehicle 0.4 ± 0.5 mmHg; *p* < 0.05; Figures 2, 3A). The injection of L-glutamate did not elicit significant alteration in RSNA, DIA burst amplitude and fR in NT rats (ΔRSNA: 28.8 ± 5.2% vs. vehicle −0.3 ± 1.1%; ΔDIA Burst Amp: −19.9 ± 15.3% vs. vehicle −0.5 ± 1.7%; ΔfR: −6.7 ± 8,2 cpm vs. vehicle −2,5 ± 1.8 cpm; Figures 2, 3C,D) and SHR (ΔRSNA: 40.0 ± 3.2% vs. vehicle 1.4 ± 0.6%; ΔDIA Burst Amp: 10.7 ± 12% vs. vehicle −3.5 ± 1.6%; ΔfR: −3.5 ± 2.4 cpm vs. vehicle −0.03 ± 1.2 cpm; Figures 2, 3B–D).

Unilateral nanoinjection of BBS increased MAP in NT rats (17.1 ± 1.7 mmHg vs. vehicle −0.7 ± 0.3 mmHg; *p* < 0.05; Figures 2A, 3A) without significant change in RSNA (45.5 ± 6.9% vs. vehicle −0.3 ± 1.1%; Figures 2A, 3B), DIA burst amplitude (21.3 ± 6.1% vs. vehicle −0.5 ± 1.7%; Figures 2A, 3C) and fR (−0.1 ± 3.7 cpm vs. vehicle −2.5 ± 1.8 cpm; Figures 2A, 3D). In SHR, unilateral nanoinjection of BBS increased MAP (28.8 ± 3.2 mmHg vs. vehicle 0.3 ± 0.4 mmHg; *p* < 0.05; Figures 2B, 3A) without significant alteration in RSNA (31.8 ± 3.7% vs. vehicle 1.4 ± 0.6%; Figures 2B, 3B) and fR (11.9 ± 3.5 cpm vs. vehicle 0.0 ± 1.2 cpm; Figures 2B, 3D). Unilateral nanoinjection of BBS enhanced DIA burst amplitude (81.2 ± 37.9% vs. vehicle −3.5 ± 1.5%; *p* < 0.05; Figures 2B, 3C).

In NT rats, bilateral nanoinjection of BBS increased MAP (33.9 ± 6.6; 27.8 ± 2.9; 16.8 ± 3.4 mmHg vs. vehicle −0.7 ± 0.3 mmHg at 1, 5, and 15 min, respectively; *p* < 0.05; Figures 2A, 3A) and RSNA (97.8 ± 12.9; 73.1 ± 23.2; 66.9 ± 30.2% vs. vehicle −0.3 ± 1.1%, at 1, 5, and 15 min, respectively; *p* < 0.05; Figures 2A, 3B). An increase in MAP (37.1 ± 4.6; 26.2 ± 5.2; 19.5 ± 5.5 mmHg vs. vehicle 0.3 ± 0.4 mmHg, at 1, 5, and 15 min, respectively; *p* < 0.05; Figures 2B, 3A) and RSNA (87.9 ± 18.1; 68.7 ± 16.8% vs. vehicle 1.4 ± 0.6%, at 1 and 5 min, respectively; *p* < 0.05; Figures 2B, 3B) were observed in SHR.

The DIA burst amplitude was not altered by bilateral nanoinjections of BBS in NT rats (23.2 ± 3.3; 19.3 ± 6.0; 15.3 ± 17.3% vs. vehicle −0.5 ± 1.7%; at 1, 5, and 15 min, respectively; Figures 2A, 3C). On the other hand, bilateral nanoinjections of BBS increased DIA burst amplitude in SHR (111.0 ± 25.3; 94.5 ± 23.1; 90.9 ± 27.7% vs. vehicle −3.5 ± 1.5%; at 1, 5, and 15 min, respectively; *p* < 0.05; Figures 2B, 3C). The bilateral nanoinjection of BBS did not induce significant changes in fR of NT rats (−5.1 ± 9.3; −2.6 ± 7.4 cpm; −2.4 ± 6.4 vs. vehicle −2.5 ± 1.8 cpm; at 1, 5, and 15 min, respectively; Figures 2A, 3D) and SHR (10.5 ± 9.1; 8.7 ± 8.9; 15.4 ± 9.2 cpm vs. vehicle 0.0 ± 1.2 cpm; at 1, 5, and 15 min, respectively; Figures 2B, 3D).

The unilateral nanoinjection of BBS did not elicit significant alteration in HR of NT rats (−14.5 ± 4.9 bpm vs. vehicle −3.2 ± 1.6 bpm). The bilateral nanoinjection of BBS decreased HR (−30.1 ± 7.3 bpm; −27.0 ± 6.8 bpm; −23.9 ± 6.8 bpm vs. vehicle −3.2 ± 1.6 bpm; at 1, 5, and 15 min, respectively; *p* < 0.05). In SHR, the unilateral (18.4 ± 3.8 bpm vs. vehicle −8.5 ± 1.9 bpm; *p* < 0.05) and bilateral nanoinjections of BBS (26.3 ± 7.0 bpm; 27.4 ± 7.7 bpm; 25.8 ± 8.2 bpm vs. vehicle −8.5 ± 1.9 bpm; at 1, 5, and 15 min; respectively; *p* < 0.05) increased HR.

### Synchronization of sympathetic discharge during the respiratory cycle after bilateral nanoinjection of BBS into RVLM

**Figure 4:**
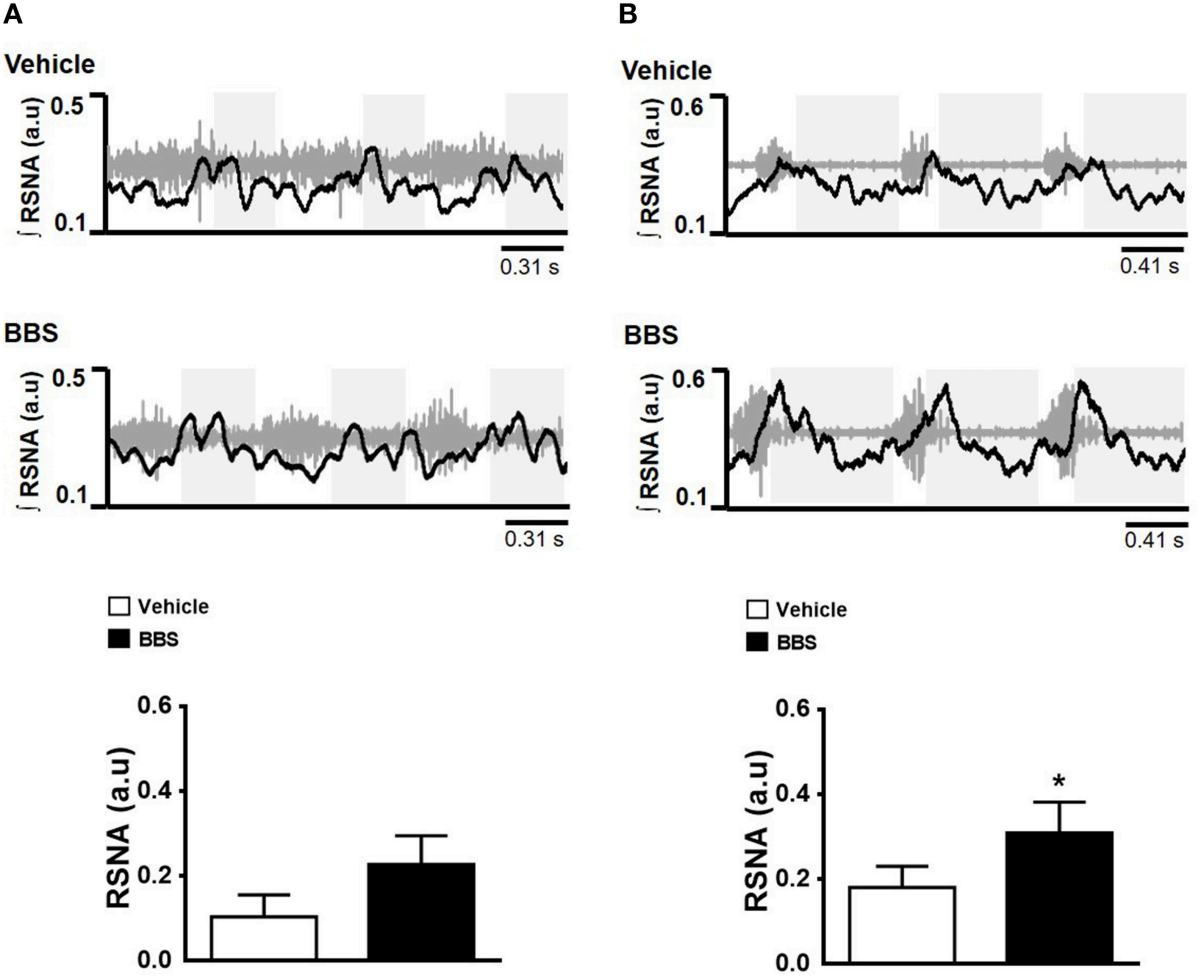
**Effects of bilateral nanoinjection of bombesin into RVLM on respiratory synchronization of RSNA**. Respiratory modulation after bilateral nanoinjection of vehicle (150 mM NaCl) and bombesin (BBS; 0.3 mM) into RVLM of normotensive **(A)** and hypertensive rats **(B)**. Examples show three respiratory cycles. The gray boxes show the expiratory phase. Maximum amplitude of renal sympathetic nerve activity (RSNA) during post-inspiratory are present in the graphics. ^*^Different from vehicle. *p* < 0.05; *n* = 7.

The BBS nanoinjected into RVLM elicited an increase in post-inspiratory RSNA peak in SHR (0.309 ± 0.07 a.u vs. vehicle 0.180 ± 0.05 a.u.; *p* < 0.05; Figure 4B) but not in NT rats (0.227 ± 0.06 a.u vs. vehicle 0.103 ± 0.05 a.u.; Figure 4A).

### Cardiorespiratory and sympathetic changes produced by BB_1_ receptor blockade in the RVLM neurons in NT rats and SHR

**Figure 5:**
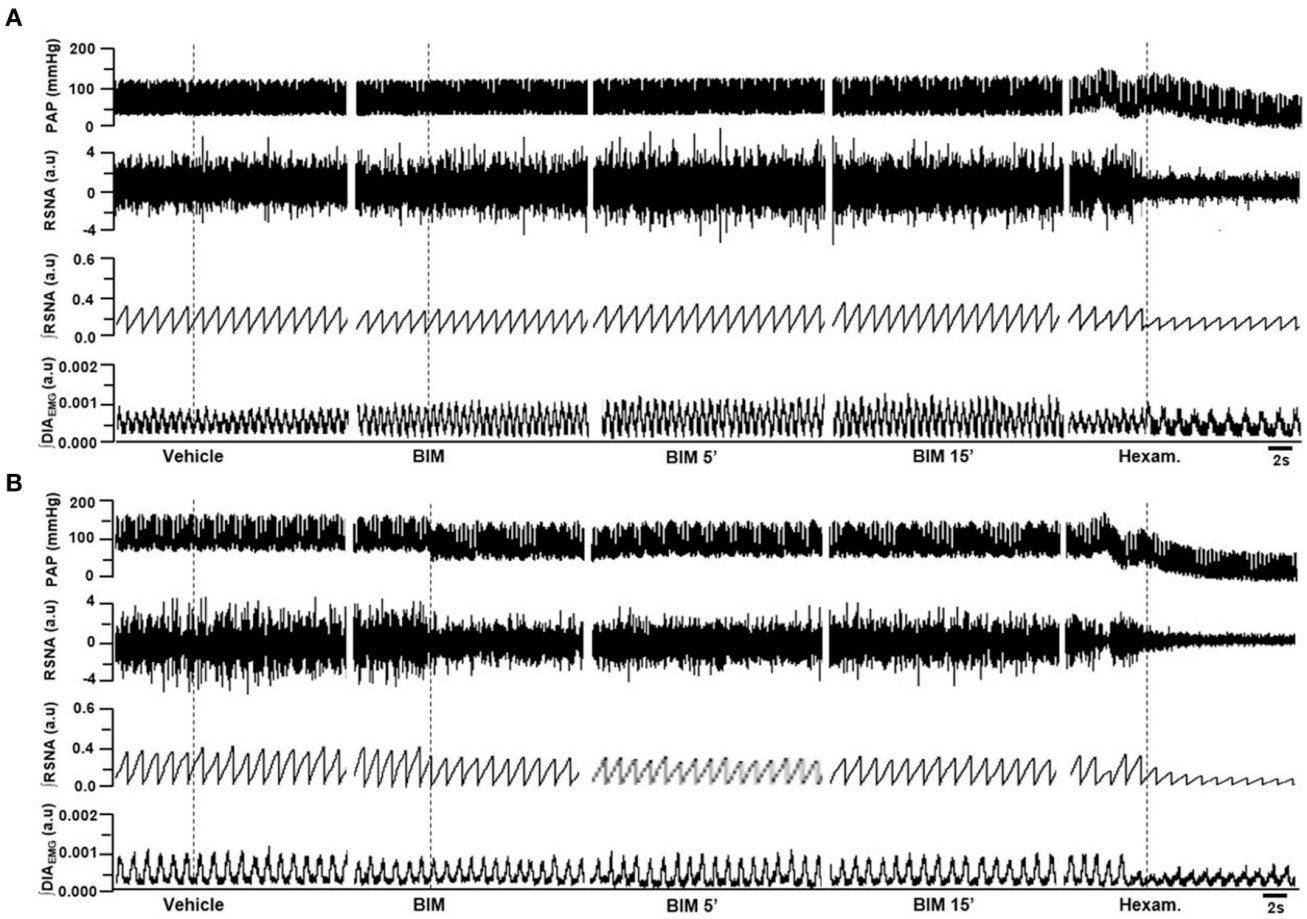
**Examples showing the cardiorespiratory and sympathetic effects induced by BB_**1**_ receptors blockade in the RVLM**. Digitized record of cardiovascular, sympathetic and respiratory responses produced by vehicle (150 mM NaCl) and bilateral nanoinjection of BIM-23127 (3 mM; BB_1_ receptor antagonist; 1, 5, and 15 min after BIM) into RVLM in normotensive **(A)** rats and hypertensive rats **(B)**. Pulsatile arterial pressure (PAP), renal sympathetic nerve activity (RSNA), integrate of renal sympathetic nerve activity (∫RSNA) and integrate of diaphragm motor activity (∫ DIA_EMG_) and hexam (hexamethonium).

**Figure 6:**
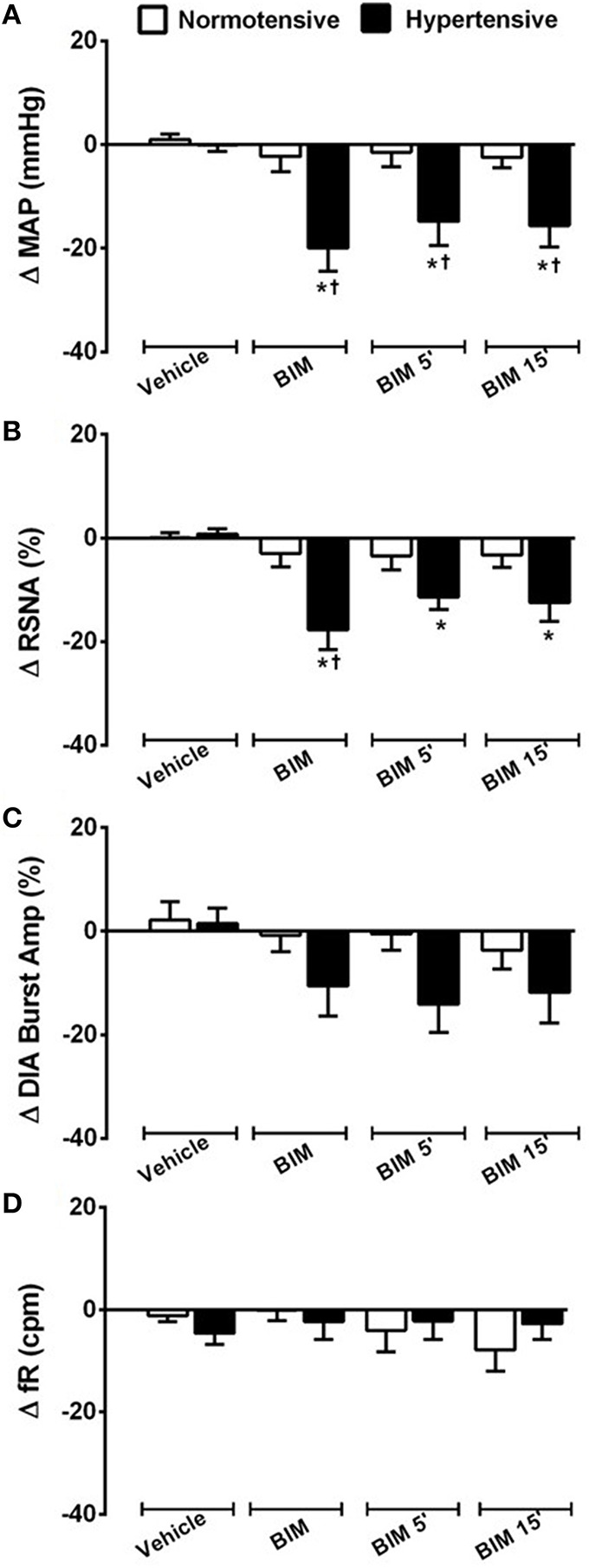
**Cardiovascular, sympathetic, and respiratory responses induced by BB_**1**_ receptors blockade into RVLM**. Changes produced by bilateral nanoinjecton of vehicle (150 mM NaCl) and BIM-23127 (3 mM; BB_1_ receptor antagonist; 1, 5, and 15 min) into RVLM on mean arterial pressure (MAP; **A**), renal sympathetic nerve activity (RSNA; **B**), diaphragm burst amplitude (DIA Burst Amp; **C**), and respiratory frequency (fR; **D**) in normotensive and hypertensive rats. ^*^different from vehicle; ^†^different from normotensive rats. *p* < 0.05; *n* = 6.

The bilateral nanoinjection of BIM-23127 into RVLM did not produce significant changes in MAP (−2.2 ± 2.8; −1.4 ± 2.7; −2.4 ± 1.9 mmHg vs. vehicle 1.0 ± 1.0 mmHg, at 1, 5, and 15 min, respectively; Figures 5A, 6A), RSNA (−2.9 ± 2.5; −3.4 ± 2.7; −3.3 ± 2.3% vs. vehicle 0.0 ± 0.9% at 1, 5, and 15 min, respectively; Figures 5A, 6B), DIA burst amplitude (−0.7 ± 3.5; −0.5 ± 3.1; −3.7 ± 3.5% vs. vehicle 2.1 ± 3.5% at 1, 5, and 15 min, respectively; Figures 5A, 6C) and fR (−0.1 ± 1.9; −4.0 ± 4.2; −7.8 ± 4.1 cpm vs. vehicle −1.1 ± 1.1 cpm at 1, 5, and 15 min, respectively; Figures 5A, 6D) in NT rats.

In SHR, bilateral nanoinjection of BIM-23127 reduced MAP (−19.9 ± 4.4; −14.8 ± 4.6; −15.6 ± 4.1 mmHg vs. vehicle −0.0 ± 1.2 mmHg, at 1, 5, and 15 min respectively; *p* < 0.05; Figures 5B, 6A) and RSNA (−17.7 ± 3.8; −11.3 ± 2.4; −12.4 ± 3.5% vs. vehicle 0.7 ± 1.0% at 1, 5, and 15 min, respectively; *p* < 0.05; Figures 5B, 6B). No significant changes were observed in the DIA burst amplitude (−10.5 ± 5.9; –14.0 ± 5.4; −11.8 ± 5.9% vs. vehicle 1.5 ± 2.9% at 1, 5, and 15 min, respectively; Figures 5B, 6C) and fR (−2.3 ± 3.5; −2.2 ± 3.5; −2.7 ± 3.0 cpm vs. vehicle −4.6 ± 2.2 cpm at 1, 5, and 15 min, respectively; Figures 5B, 6D) in SHR.

The blockade of BB_1_ receptor did not alter HR in both NT rats (−3.8 ±3.0 bpm; −4.9 ± 2.4 bpm; −7.8 ± 2.6 bpm vs. vehicle 1.9 ± 1.6 bpm, at 1, 5, and 15 min, respectively) and SHR (−11.5 ±6.7 bpm; −6.1 ± 5.0 bpm; −5.7 ± 6.2 bpm vs. vehicle 0.4 ± 1.2 bpm, at 1, 5, and 15 min, respectively).

### Cardiorespiratory and sympathetic responses to BBS injected after blockade of RVLM BB_1_ receptors

Table 2 shows the cardiorespiratory and sympathetic changes induced by unilateral nanoinjection of BBS into RVLM before and after injection of BIM-23127 (BB_1_ receptor antagonist) in NT rats and SHR. In both groups, the BBS-induced increase in MAP and RSNA was abolished by BIM-23127. The significant changes in BBS-induced DIA burst amplitude was abolished by the blockade of BB_1_ receptors SHR.

**Table 2:** **Changes in the mean arterial pressure (MAP), renal sympathetic nerve activity (RSNA) and diaphragm burst amplitude (DIA burst Amp) induced by unilateral injection of bombesin (BBS; 0.3 mM) into RVLM before and after BB_**1**_ receptor blockade with BIM-23127 (3 mM) in normotensive (NT) and spontaneously hypertensive (SHR) rats**.

**Group**	**Nanoinjections**	**Δ MAP (mmHg)**	**Δ RSNA (%)**	**Δ DIA burst Amp (%)**
NT	BBS before BIM-23127	25.2 ± 0.5	47.3 ± 9.1	20.1 ± 8.7
	BBS after BIM-23127	1.5 ± 0.5^*^	2.1 ± 0.9^*^	−5.9 ± 7.2
SHR	BBS before BIM-23127	24.8 ± 2.9	41.6 ± 4.6	24.6 ± 5.5
	BBS after BIM-23127	6.5 ± 0.6^*^	4.7 ± 1.0^*^	−0.1 ± 4.3^*^

*Values are expressed as mean ± SEM*.

* *different from BBS before BIM-23127; p < 0.05. n = 3–6*.

## Discussion

Previous studies have shown that BBS increases blood pressure (Brown and Guyenet, 1985; Zogovic and Pilowsky, 2011) and sympathetic tone (Zogovic and Pilowsky, 2011). However, these studies did not established the effects of BBS in the brainstem and their role in the maintenance of hypertension. In the present study, we provided the first evidence that BBS acting in RVLM elicits a significant increase in MAP and RSNA of both NT rats and SHR. In addition, the administration of BBS into RVLM increased DIA burst amplitude and post-inspiratory RSNA burst in SHR. The blockade of BB_1_ receptors in the RVLM reduces MAP and RSNA in SHR but not in NT rats. These results strongly indicate that the sympathoexcitation associated with pressor response to BBS administration is mediated by BB_1_ receptors that are located on RVLM neurons. It has been suggested that tonic activation of BB_1_ receptors is involved in the maintenance of high arterial pressure in SHR.

Zogovic and Pilowsky (2011), showed a long-lasting increase in the splanchnic SNA (sSNA), blood pressure and phrenic nerve amplitude by the intrathecal injection of BBS. Glazkova and Inyushkin (2006) reported that microinjection of BBS in the solitary tract nucleus (NTS) stimulated respiration, and increased the level of pulmonary ventilation, respiratory volume, and bioelectrical activity of the inspiratory muscles. Our results showed that BBS injected into the RVLM increased arterial blood pressure and sympathetic vasomotor tone in both NT and SHR. This result suggests a modulatory action of BBS in RVLM neurons.

Recently, Li et al. (2016) showed that small neural subpopulation is involved in the control of breathing. The retrotrapezoid nucleus/parafacial respiratory group (RTN/pFRG) expresses neuromedin B (BBS-like peptide genes) and GRP. This neural subpopulation project to preBötzinger Complex (preBötC, the respiratory rhythm generator that expresses neuromedin B and GRP receptors). The vasomotor presympathetic neurons in all anteroposterior extension of RVLM are intercalated with ventral respiratory column neurons (Smith et al., 2007; Alheid and McCrimmon, 2008; Wang et al., 2009). As a result of the proximity with respiratory neurons, the BBS injection into the RVLM could activate neuromedin B or GRP receptors and contribute to the increase in respiratory drive that was observed in SHR.

The RVLM neurons play essential role in the generation of sympathetic outflow (Guertzenstein, 1973; Guyenet, 2006; Wang et al., 2009) and regulation of peripheral chemosensitive and barosensitive sympathetic efferents (Sun and Reis, 1995; Miyawaki et al., 1996; Dampney et al., 2002; Alheid and McCrimmon, 2008). The neuronal activity of the RVLM is determined by the action of excitatory and inhibitory synapses that involve glutamate and γ-aminobutyric acid (GABA) neurotransmitters, respectively (Sun and Reis, 1995; Miyawaki et al., 1996; Ito et al., 2000; Schreihofer et al., 2000; Alheid and McCrimmon, 2008). In addition, some neuropeptides have been reported to play important modulatory role in the integration cardiovascular responses (Ito et al., 2000; Alheid and McCrimmon, 2008; Abbott and Pilowsky, 2009).

Several studies have shown that the activation or blockade of neuropeptide receptors could cause a long-term response (Abbott and Pilowsky, 2009; Zogovic and Pilowsky, 2011). The nature of response can be explained (partly) by the receptor-ligand type. For instance, the sensitization of G-protein coupled receptors are related to a wide range of intracellular event cascades such as changes in ion channel permeability, activation of kinases, and protein phosphorylation (Springell et al., 2005a,b).

Zogovic and Pilowsky (2011) reported that intrathecal injection of BBS elicited gradual increase in MAP and splanchnic sympathetic nerve activity (sSNA) within 5 min. This increase returned to control level after approximately 35 min of BBS injection. In our study, we showed that BBS injection into RVLM caused a rapid increase in blood pressure. This increase persisted during 15 min of bilateral nanoinjection of BBS. The injection of BIM induced rapid decrease in the MAP and RSNA (the decrease was maintained during 15 min). Some authors have reported instantaneous response to BBS administration (Erspamer et al., 1972; Chahl and Walker, 1981; Bayorh and Feuerstein, 1985; Kaczynska and Szereda-Przestaszewska, 2011). Intravenous administration of BBS caused immediate increase in MAP of anesthetized animals (Erspamer et al., 1972; Kaczynska and Szereda-Przestaszewska, 2011). According to the author, this cardiovascular response appeared to be mediated via α-adrenergic receptors (Kaczynska and Szereda-Przestaszewska, 2011).

Experimental evidences have demonstrated that an increase in RVLM neuronal activity could contribute to the development and maintenance of hypertension in SHR (Smith and Barron, 1990; Yang et al., 1995; Matsuura et al., 2002; Ito et al., 2003). The bicuculline (GABAa receptor antagonist) injection into RVLM slightly increased the arterial blood pressure in SHR when compared to NT rats. This result suggest excessive excitatory drive of RVLM pre-sympathetic neurons in hypertensive rats (Smith and Barron, 1990). The inhibition of glutamatergic neurotransmission by kynurenic acid (KYN) injection into RVLM of SHR decreased the arterial pressure (Ito et al., 2000). Matsuura et al., (2002), showed that basal membrane potential in irregularly firing RVLM neurons is less negative in neonatal SHR. In consequence, the RVLM neurons in these animals are more easily excitable. The firing rate is faster in neonatal SHR when compared with NT rats. In our study, we demonstrated that the blockade of RVLM BB_1_ receptors decreased MAP and RSNA in SHR, but not in NT rats. These findings suggest that the activation of BB_1_ receptors in the RVLM could have contributed to the maintenance of high arterial pressure in SHR.

Our findings are consistent with previous studies, which showed a decrease blood pressure of anesthetized hypertensive rats as a result of pharmacological blockade of RVLM neurons (Bergamaschi et al., 1999, 2014; Ito et al., 2000, 2003; Suhaimi et al., 2010; Du et al., 2013). The BBS injection into RVLM, in the present study, induced similar increase in the MAP and RSNA of SHR when compared with NT rats. The anesthesia reduced MAP in hypertensive rats more than in NT controls. Unlike non-anesthetized rats, the subjection of rats to anesthesia is assumed to have prevented the some responses to BBS.

Our results showed that BBS injection into RVLM induced sympathoexcitation with an increase in the blood pressure of NT rats and SHR. This effect indicates that RVLM could integrate neuronal pathway that are involved in BBS induced cardiorespiratory and sympathetic effects. The blockade of BB_1_ receptors in the RVLM of SHR, but not NT rats, decreased MAP and RSNA. This result suggests that the tonic activation of BB_1_ receptors is involved in the maintenance of high blood pressure in anesthetized SHR. The changes in the cardiorespiratory and sympathetic activity as a result of an activation or inhibition of RVLM BBS receptors suggests an important cardiorespiratory role of BBS and related peptides.

## Author contributions

Conceived and designed the experiments: GP, CD, AF, AD, AR, DC. Performed the experiments: ID, AM, SM, AC, KG. Analyzed the data: ID, AM, ED, MF, JF, DC, EC, GP. Contributed reagents/materials/analysis tools: DC, CD, DR, EC, AF, AD, AR, GP. Wrote the paper: ID, AM, ED, MF, JF, DR, EC, AF, AR, GP.

### Conflict of interest statement

The authors declare that the research was conducted in the absence of any commercial or financial relationships that could be construed as a potential conflict of interest.
